# Cross-Talk Between Butyric Acid and Gut Microbiota in Ulcerative Colitis Following Fecal Microbiota Transplantation

**DOI:** 10.3389/fmicb.2021.658292

**Published:** 2021-04-12

**Authors:** Hao-Ming Xu, Hong-Li Huang, Jing Xu, Jie He, Chong Zhao, Yao Peng, Hai-Lan Zhao, Wen-Qi Huang, Chuang-Yu Cao, Yong-Jian Zhou, You-Lian Zhou, Yu-Qiang Nie

**Affiliations:** Department of Gastroenterology and Hepatology, Guangzhou Digestive Disease Center, Guangzhou First People’s Hospital, School of Medicine, South China University of Technology, Guangzhou, China

**Keywords:** butyric acid, gut microbiota, ulcerative colitis, gastroenterology and hepatology, fecal microbiota transplantation

## Abstract

Fecal microbiota transplantation (FMT) can inhibit the progression of ulcerative colitis (UC). However, how FMT modulates the gut microbiota and which biomarker is valuable for evaluating the efficacy of FMT have not been clarified. This study aimed to determine the changes in the gut microbiota and their relationship with butyric acid following FMT for UC. Fecal microbiota (FM) was isolated from healthy individuals or mice and transplanted into 12 UC patients or colitis mice induced by dextran sulfate sodium (DSS). Their clinical colitis severities were monitored. Their gut microbiota were analyzed by 16S sequencing and bioinformatics. The levels of fecal short-chain fatty acids (SCFAs) from five UC patients with recurrent symptoms after FMT and individual mice were quantified by liquid chromatography–mass spectrometry (LC–MS). The impact of butyric acid on the abundance and diversity of the gut microbiota was tested *in vitro.* The effect of the combination of butyric acid-producing bacterium and FMT on the clinical responses of 45 UC patients was retrospectively analyzed. Compared with that in the controls, the FMT significantly increased the abundance of butyric acid-producing bacteria and fecal butyric acid levels in UC patients. The FMT significantly increased the α-diversity, changed gut microbial structure, and elevated fecal butyric acid levels in colitis mice. Anaerobic culture with butyrate significantly increased the α-diversity of the gut microbiota from colitis mice and changed their structure. FMT combination with *Clostridium butyricum*-containing probiotics significantly prolonged the UC remission in the clinic. Therefore, fecal butyric acid level may be a biomarker for evaluating the efficacy of FMT for UC, and addition of butyrate-producing bacteria may prolong the therapeutic effect of FMT on UC by changing the gut microbiota.

## Introduction

Ulcerative colitis (UC) is a heterogeneous, chronic and inflammatory disease. Pathologically, UC is derived from environmental risk factors and gut microbiota disturbance that lead to the imbalance of mucosal immune responses in genetically susceptible individuals (Kobayashi et al., 2020). Fecal microbiota transplantation (FMT) is an emerging novel therapy for UC. The FMT procedure includes isolating fecal microbiota (FM) from healthy donors and transplanting into the patient’s gastrointestinal tract to reconstruct the gut microbiota and restore microbial homeostasis (Antushevich, 2020). FMT targets gut microbiome dysbiosis, and despite its unappealing nature, FMT has been demonstrated to induce remission of moderate–severe active UC in humans (Cui et al., 2015; Lai et al., 2019; Chen et al., 2020). Furthermore, FMT can maintain long-term remission in some UC patients (Sood et al., 2019). In addition, FMT is a cost-effective, safe and effective therapy for some UC patients (Kelly et al., 2015; Marcella et al., 2021). Our previous study has shown that FMT benefits UC patients at least for 3 months (Chen et al., 2020). Hence, further discovery of new biomarkers for evaluating the efficacy of FMT therapy for UC patients and modification of FMT to achieve long-term therapeutic efficacy will be of high significance.

Short-chain fatty acids (SCFAs), such as acetic, propionic, and butyric acids, are end products of non-starch polysaccharides by the gut bacterial fermentation. Functionally, butyric acid is a main energy source for the colonic mucosa and can increase the expression of tight junction proteins to support the integrity of the gut barrier. A previous study has shown that increased levels of intestinal butyric acid ameliorate mucosal inflammation and oxidative stress in rodents (Parada Venegas et al., 2019). Given that many types of bacteria can produce butyric acid and other SCFAs, we hypothesize that FMT can modulate the abundance of butyrate-producing bacteria and increase fecal butyric acid contents, contributing to its therapeutic efficacy in inducing long-term remission of active UC.

In this study, we analyzed the fecal samples from FMT-treated UC patients for their gut microbiota and fecal butyric acid contents by 16S sequencing and metabonomic analysis, respectively. We further established dextran sulfate sodium (DSS)-induced colitis mouse model, and following FMT with human or mouse microbiota, we analyzed the abundance and diversity of the gut microbiota and fecal SCFA contents in these mice. Moreover, we tested the impact of butyrate on the abundance and diversity of anaerobically cultured microbiota from healthy and colitis mice. Finally, we retrospectively analyzed the therapeutic effect of the combination of butyric acid-producing bacterium and FMT therapy on the clinical activity of 45 UC patients. Our data suggest that fecal butyric acid level may be a biomarker for evaluating the efficacy of FMT for UC and addition of butyrate-producing bacteria may prolong the therapeutic effect of FMT on UC by changing the gut microbiota.

## Materials and Methods

### Experimental Design and Technical Operation

#### Intervention of FMT and Collection of Human Fecal Samples

Fresh feces (150–200 g each) from a healthy donor were dissolved in 1,000 ml of physiological saline, and the contained microbiota were isolated using the GenFMTer automatic purification system (FMT Medical) according to the manufacturer’s protocol. At 1 day post-TET insertion, individual patients were administrated with 150 ml of physiological saline containing ∼50 cm^3^ centrifuged microbiota into their entire colon *via* the TET tube. After FMT, patients were required to remain in the right lateral position for ≥ 30 min and were allowed to eat 2 h later. The FMT procedure was repeated every other day for 2–3 treatments (Cui et al., 2015). A total of 12 UC patients were transplanted with FM from a healthy donor from January 2017 to December 2017, as a previous study (Chen et al., 2020). Those 12 patients did not receive any antibiotics, probiotics, hormones, and other drugs for at least 3 months pre-FMT and post-FMT. Their fecal samples were collected by patients 1 week before and 1 and 3 months after the FMT and immediately stored at –80°C in the biological sample bank of our hospital (approval no. K-2017-103-02). Their demographic and clinical characteristics, including defecation number, bloody stool, Mayo scores, and laboratory testing serum C-reactive protein (CRP), procalcitonin (PCT), and erythrocyte sedimentation rate (ESR) levels, were recorded at each time point (Supplementary Tables 1, 2). There were 5 out of 12 UC patients with recurrent symptoms after FMT treatment. Their fecal samples were collected for 16S sequencing and targeted metabonomics.

#### Establishment of a Mouse Model of DSS-Induced Colitis

Male BALB/c mice at 6–8 weeks old were purchased from Guangdong Medical Laboratory Animal Center (GDMLAC; certificate number SYXK 2013-0002, Foshan, China). The mice were housed in a specific pathogen-free room with a 12-h light/dark cycle, consistent temperature (24°C), and humidity (50–70%) and allowed free access to food and water. The experimental procedures were approved by the Animal Ethics Committee of Guangzhou First People’s Hospital (acceptance no. 2017-202).

**FIGURE 1:**
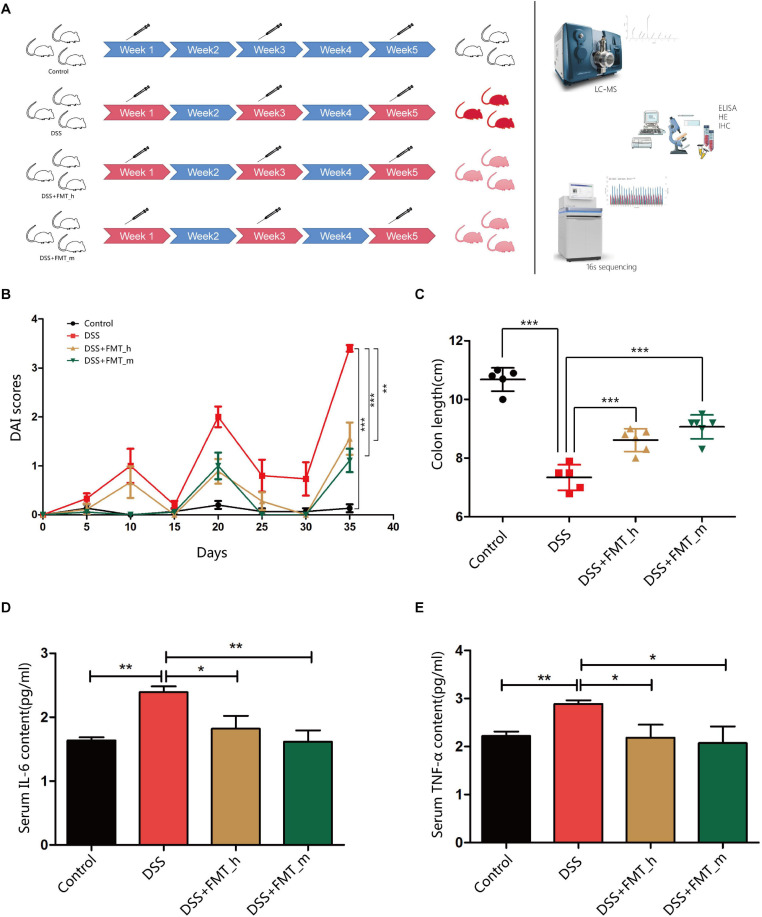
FMT with the gut microbiota from healthy donors or mice significantly ameliorates clinical symptoms and inflammation in colitis mice. BALB/c mice were randomized and fed with normal water as the control or water containing 2.5% DSS to induce colitis. The control and some DSS-fed mice (DSS group, *n* = 6) were fed with PBS, whereas other DSS-fed mice were fed with fresh human or mouse fecal microbiota as the DSS + FMT_h or DSS + FMT_m group (*n* = 6 per group). **(A)** Schematic diagram of the experimental design. The degrees of red colors indicate the severity of colitis. **(B)** The dynamics of Disease Activity Index (DAI) in each group. **(C)** Mouse colon lengths in each group. **(D)** The levels of serum IL-6. **(E)** The levels of serum TNF-α. Data are individual means or the mean ± SD of each group from three separate experiments. *p < 0.05, **p < 0.01, ***p < 0.001.

The mice were randomized and fed with regular water as the control or water containing 2.5% DSS (Sigma, United States) every other week for 5 weeks to induce colitis (Wirtz et al., 2007; Jiang et al., 2020). The mice were divided into four groups: the control, the DSS colitis model, the human FMT, and the mouse FMT groups. The control (*n* = 5) and DSS colitis model groups (*n* = 6) were administrated with phosphate buffered saline (PBS, 0.1 ml/10 g) by gavage. The human FMT group was fed with fresh human FM (0.1 ml/10 g) prepared from the same healthy donor, whereas the mouse FMT group was fed with fresh mouse FM prepared from healthy mice (*n* = 6 per group). The experimental protocols were illustrated in the schematic diagram (Figure 1A). The mice were monitored daily for their body weights, stool consistency, and the presence of blood in the anus or stool. At the end of the experiment, their blood and stool samples were collected, and their colon tissues were dissected. The colon lengths were measured, and the dissected tissues were fixed overnight in 10% neutral buffered formalin. Their serum samples were prepared for ELISA assays.

#### Disease Activity Index Scores

The Disease Activity Index (DAI) of individual mice was scored every 5 days according to a modified version of a previously described method (Murthy et al., 1993), which included the following parameters: A) percentage of body weight loss (0: none, 1: 1–5%, 2: 5–10%, 3: 10–15%, and 4: > 15%), B) stool consistency (0: normal, 1: pasty and not sticking to the anus, 2: pasty and slightly sticking to the anus, 3: pasty and stuck to the anus, and 4: watery), and C) rectal bleeding [0: hemoccult (–), 1: hemoccult (±), 2: hemoccult (+), 3: hemoccult (++), and 4: obvious blood in stool].

#### Quantification of Serum IL-6 and TNF-α by ELISA Assays

The levels of serum interleukin 6 (IL-6) and tumor necrosis factor α (TNF-α) were quantified using the mouse IL-6 (E03I0006) and TNF-α (E03T0008) ELISA kits according to the manufacturer’s instructions (BlueGene, Shanghai, China). The sample absorbance was measured in a microplate reader (Multiskan MK3; Thermo Fisher Scientific, Waltham, MA, United States).

#### Histological Evaluation of Colitis

The mice were euthanized, and their entire colon tissues from the cecum to the anus were dissected. The colon tissues were opened longitudinally along the mesenteric border, and its contents were washed with cold saline. The clean tissue samples were fixed in buffered formalin and embedded in paraffin. The tissue sections (5 μm) were stained with hematoxylin and eosin (H&E). The sections were evaluated histopathologically by two independent investigators in a blinded manner, based on (A) the degrees of inflammation (0: no inflammatory infiltrate, 1: infiltrates in the lamina propria, 2: infiltrates in the submucosa, and 3: transmural infiltration), (B) ulceration (0: no ulceration, 1: one or two ulcers, 2: three or four ulcers, and 3: more than four ulcers), (C) mucosal hyperplasia (0: normal, 1: slightly thickened mucosa with minimal fibrosis, 2: mucosal thickening with fibrous hyperplasia, and 3: extensive mucosal thickening and fibrous hyperplasia or granulation), and (D) edema (0: none, 1: 0–30%, 2: 30–70%, and 3: >70%) (Lin et al., 2020).

#### Evaluation of Intestinal Barrier Function by Immunohistochemistry

The levels of ZO-1 and occludin expression in individual colon tissue samples were examined by immunohistochemistry (IHC) using anti-ZO-1 (ab216880) and anti-occludin (ab168986) from Abcam (Cambridge, MA, United States) and the MaxVisiton kit (Maixin Biol, Fuzhou, Fujian, China) (Kato et al., 2007). The sections were independently evaluated by two pathologists in a blinded manner. Protein expression was evaluated according to the extension and intensity of staining and scored as the extension of 0: 0–5%, 1: 6–25%, 2: 26–50%, 3: 51–75%, and 4: 76–100% of cells stained positively and the staining intensity of 0: no staining, 1: weak staining, 2: moderate staining, and 3: strong staining. A final score was generated by multiplying the staining extension score by the staining intensity score and used for stratification of low expression (a final score < 6) and high expression (a final score ≥ 6).

#### Measurements of Fecal SCFAs by Liquid Chromatography–Mass Spectrometry

Acid solutions of 200 mM 3-nitrobenzene trap (3-NPH) and 120 mM (6% pyridine) 1-(3-dimethylaminopropyl)-3-ethylcarbodiimide hydrochloride (EDC) were prepared with 50% acetonitrile aqueous. Individual fecal samples (100 mg each) were mixed with 800 μl of deionized water and vortexed for 30 s. The fecal samples were grinded for 300 s at 60 Hz and centrifuged at 13,780 × *g* at 4°C for 15 min. Subsequently, 400 μl of supernatants was collected and mixed with 3-NPH and EDC (2:1:1). After the reaction for 60 min, the samples were centrifuged, diluted 100 times, and subjected to liquid chromatography–mass spectrometry (LC–MS) analyses as described (Han et al., 2015) in a Waters Acquity UPLC/AB SCIEX 5500 QQQ LC–MS system.

#### Fecal DNA Extraction and Gut Microbiota Analysis

Total fecal DNA was extracted using the HiPure Stool DNA Kit (Magen, Guangzhou, China) according to the manufacturer’s protocol. The 16S rDNA target regions of individual ribosomal RNA genes of different microbiota were amplified by PCR (94°C for 2 min; 30 cycles of 98°C for 10 s, 62°C for 30 s, and 68°C for 30 s; and a final extension at 68°C for 5 min) (Guo et al., 2017). The PCR reactions were performed in triplicate using a 50 μl mixture containing 5 μl of 10 × KOD buffer, 5 μl of 2 mM deoxynucleotide triphosphates (dNTPs), 3 μl of 25 mM MgSO_4_, 1.5 μl of each primer (10 μM), 1 μl of KOD polymerase, and 100 ng of template DNA. The PCR reagents were purchased from TOYOBO, Japan. The sequences of primers were 341F: CCTACGGGNGGCWGCAG and 806R: GGACTACHVGGGTATCTAAT (Guo et al., 2017). The amplicons were resolved on 2% agarose gels and purified using the AxyPrep DNA Gel Extraction Kit (Axygen Biosciences, Union City, CA, United States) according to the manufacturer’s instruction, followed by quantifying using the ABI StepOnePlus Real-Time PCR System (Life Technologies, Foster City, United States). The purified amplicons in equimolar were pooled and paired-end sequenced (PE250) on an Illumina platform.

#### Anaerobic Culture of the Butyrate–Feces Mixture *in vitro*

The colitis and control mice (*n* = 5 per group) were anesthetized with excessive pentobarbital sodium, and their colon fecal samples were collected in an anaerobic environment (Figure 6A). Individual fecal samples (50 mg each) were mixed with 1 ml of PBS, which were injected into the anaerobic blood culture bottle (BD BACTEC lytic/10 anaerobic, New Zealand) or the bottle containing 0.1% sodium butyrate (pH 7.2–7.4), followed by cultured for 48 h at 37°C (Lagier et al., 2016, 2018). The growing bacteria were collected by centrifugation. Their total DNA was extracted, and the 16S rDNAs were sequenced.

#### Retrospective Analysis of UC Patients Who Received FMT Alone or Combined With *Clostridium butyricum*-Containing Probiotics

A total of 45 UC patients (including 12 UC patients mentioned in the Intervention of FMT and Collection of Human Fecal Samples section) received FMT from January 2017 to December 2019. In addition, 11 out of 45 UC patients took “Shiyi” (a kind of probiotics, containing abundant *C. butyricum TO-A*, *Bacillus mesentericus TO-A*, and *Streptococcus faecalis T-110*, from Toa Pharmaceutical (Japan). Those patients were stratified into the FMT-“Shiyi” group (*n* = 11) and the FMT-non-“Shiyi” group (*n* = 34). The therapeutic efficacy of individual UC patients was evaluated at 3 months post-FMT.

### Bioinformatics

#### Operational Taxonomic Units Analysis

The effective tags were clustered into operational taxonomic units (OTUs) of ≥ 97% similarity using the UPARSE (Edgar, 2013) (version 9.2.64). The most abundant tag sequence was selected as a representative sequence within each cluster. The distribution of elements among the groups was analyzed by the VennDiagram package (Chen and Boutros, 2011) (version 1.6.16) in the R project^1^. The principal components were analyzed by Principal Component Analysis (PCA) using the Vegan package (version 2.5.3) in the R project. The interested sequences were aligned using Muscle (Edgar, 2004) (version 3.8.31), and the clustering trees were constructed using UPGMA (Unweighted Pair Group Method with Arithmetic Mean) and FastTree (Price et al., 2010) (version 2.1).

#### Community Composition Analysis

The representative sequences were classified into organisms by a naive Bayesian model using the RDP classifier (Wang et al., 2007) (version 2.2) based on the SILVA (Pruesse et al., 2007) (version 132) or Greengene (DeSantis et al., 2006) (version gg_13_5) databases. The community composition was visualized as a stacked bar plot using the ggplot2 package^2^ in the R project. The species abundance was plotted using the pheatmap package (version 1.0.12) in the R project.

#### α-Diversity Analysis

All α-diversity indexes were calculated using the QIIME software (Caporaso et al., 2010) (version 1.9.1). The OTU rarefaction and the rank abundance curves were plotted using the ggplot2 package (version 2.2.1) in the R project. The difference in α-index between two groups was calculated by Welch’s t-test or Wilcoxon rank test in the R project Vegan package (version 2.5.3). The difference in α-index among three or more groups was analyzed by Tukey’s HSD test or Kruskal–Wallis H test in the R project Vegan package (version 2.5.3).

#### Indicator Species Analysis

The difference in species between two groups or among groups was analyzed by Welch’s t-test rank test or Tukey’s HSD test and Kruskal–Wallis H test in the R project Vegan package (version 2.5.3), respectively. The biomarker features in each group were screened using the labdsv package (version 2.0.1) in the R project.

### Statistical Analysis

Data are presented as percentage or mean ± standard deviation (SD). The difference between groups or among groups was analyzed by paired *t*-test, unpaired *t*-test, χ^2^ test, or Wilcoxon signed rank test and one-way ANOVA and *post hoc* Tukey’s test where applicable. All statistical analyses were performed using the SPSS software (version 23.0; IBM). *p*-Values <0.05 indicated statistical significance.

## Results

### The FMT Efficacy Is Related to the Abundance of SCFA-Producing Bacteria

To understand the mechanisms underlying the action of FMT, 12 UC patients were transplanted with FM from a healthy donor, and their fecal samples were collected longitudinally. During the follow-up period, we observed that 5 out of 12 patients achieved a significantly decreased Mayo scores at 1 month post-FMT (Supplementary Table 1) and they displayed a reduced frequency of stool without bloody stool. However, their clinical symptoms were recurrence at 3 months post-FMT (Supplementary Table 2). The fecal 16S sequencing data from 12 UC patients are shown in Supplementary Figure 1. Fortunately, those five UC patients with recurrent symptoms preserved excellent quality and sufficient quantity for metabonomic analysis. Furthermore, 16S sequencing and targeted SCFAs metabonomics analyses indicated that the donor FMT suspension in those five patients was enriched mainly with the following bacteria: *Lachnospira*, *Faecalibacterium*, *Ruminococcus_1*, *Roseburia*, *Akkermansia*, *Ruminococcaceae_UCG-002*, *Methanobrevibacter*, *Lachnospiraceae_NK4A136_group*, *Barnesiella*, *Campylobacter*, *Alistipes*, *Clostridium_sensu_stricto_1*, *Anaerostipes*, *Rum inococcaceae_NK4A214_group*, *[Ruminococcus]_torques_group*, *Ruminococcaceae_UCG-014*, *Ruminococcus_2*, and *[Eubact erium]_coprostanoligenes_group* (Supplementary Figure 2). One month after the FMT, the relative abundance increased in bacteria of *Lachnospira*, *Faecalibacterium*, *Ruminococcus_1*, *Akkermansia*, *Ruminococcaceae_UCG-002*, *Methanobrevibacter*, *Lachnospiraceae_NK4A136_group*, *Alistipes*, *Clostridium_sensu_stricto_1*, *Ruminococcaceae_ NK4A214_group*, *[Ruminococcus]_torques_group*, *Ruminococ caceae_UCG-014*, *Ruminococcus_2*, and *[Eubacterium]_ coprostanoligenes_group*. However, 3 months after the FMT, the relative abundance increased only in two bacteria, *Ruminococcus_1* and *Ruminococcaceae_NK4A214_group*. The average relative abundance of each group is shown in Supplementary Table 3. Most of the bacteria with increased relative abundance after the FMT were associated with the production of SCFAs, especially butyric acid.

### Butyric Acid Is a Potential Marker of FMT Efficacy

Next, we measured the levels of fecal SCFAs by LC–MS analysis. We found that the fecal SCFA contents in patients before the FMT were generally low, but increased after the FMT treatment. The contents of SCFAs were associated inversely with the degrees of disease severity in those patients (Total SCFAs-Mayo scores, *r* = –0.5335, *p* = 0.0405; Acetic acid-Mayo scores, *r* = –0.5450, *p* = 0.0357; Propionic acid-Mayo scores, *r* = –0.3800, *p* = 0.1623; Isobutyric acid-Mayo scores, *r* = –0.5362, *p* = 0.0393; Butyric acid-Mayo scores, *r* = –0.5321, *p* = 0.0412; Isovaleric acid-Mayo scores, *r* = –0.4806, *p* = 0.0698; Valeric acid-Mayo scores, *r* = –0.4671, *p* = 0.0792; Hexanoic acid-Mayo scores, *r* = –0.1283, *p* = 0.6486). Compared with the baseline, the fecal SCFA, such as acetic, isobutyric, and butyric acids, contents were significantly elevated at 1 month post-FMT (*p* = 0.026, *p* = 0.029, and *p* = 0.028, respectively), but they decreased at 3 months post-FMT (Supplementary Figure 3).

### FMT Improves DAI and Colon Lengths and Reduces Serum Levels of Inflammatory Cytokines in Colitis Mice

To further explore the function and mechanisms of FMT, we established DSS-induced colitis in mice, and following transplantation with human or mouse microbiota (Figure 1A), we monitored the dynamic process of colitis in the different groups of mice (Figure 1B). In comparison with that in the control group, the DAI values significantly increased in a fluctuated manner in colitis mice, and the DAI values in the DSS + FMT_h and DSS + FMT_m groups were significantly lower than that in the DSS group at the end of the experiment (*p* = 0.0027 and *p* = 0.0002, respectively). Similarly, the colon lengths in the DSS + FMT_h and DSS + FMT_m groups were significantly longer than that in the DSS group (*p* = 0.0006 and *p* < 0.0001, respectively, Figure 1C). The levels of serum IL-6 and TNF-α in the DSS group significantly increased (*p* = 0.0011 and *p* = 0.0022, respectively) relative to that in the control, whereas they were significantly lower in the DSS + FMT_h and DSS + FMT_m groups than in the DSS group (IL-6, *p* = 0.0467 and *p* = 0.0067; TNF-α, *p* = 0.0494 and *p* = 0.0162, respectively, Figures 1D,E).

### FMT Improves Colonic Pathological Damage and Enhances the Expression of Colonic Barrier Proteins in Colitis Mice

**FIGURE 2:**
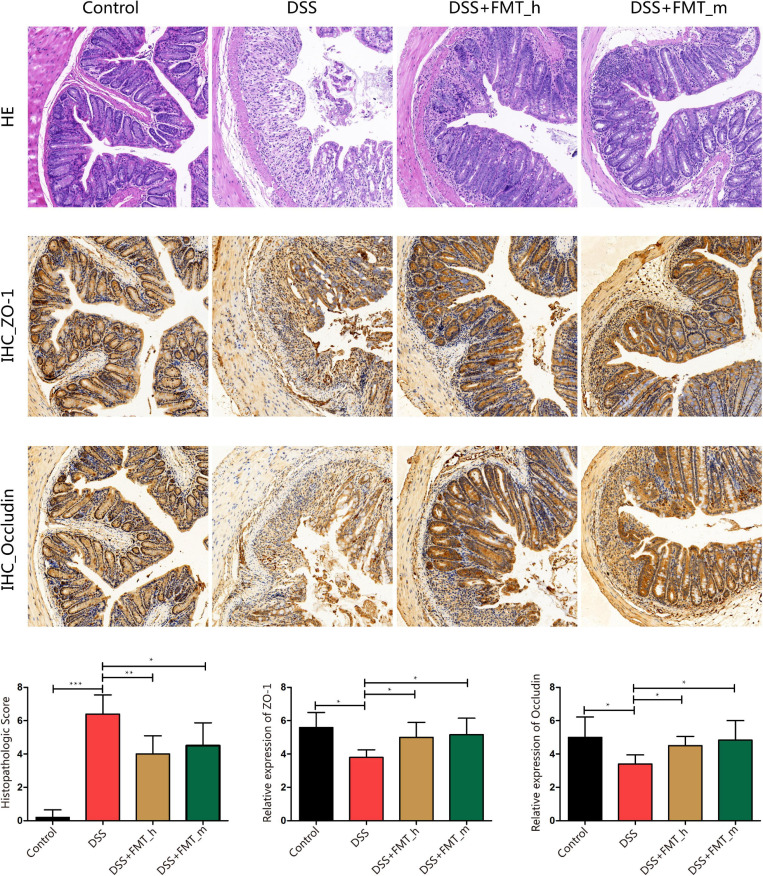
FMT mitigates the pathological changes in the colonic epithelium and rescues ZO-1 and occludin expression in the colon tissues of colitis mice. At the end of the experiment, the colonic tissues were dissected and stained with H&E or immunohistochemistry using anti-ZO-1 or anti-occludin. Data are representative images (magnification × 200) or expressed as mean ± SD of each group (*n* = 5–6) from three separate experiments. The bottom row displays colon histopathological score, relative ZO-1, and occludin expression in the colonic tissues of each group of mice. *p < 0.05, **p < 0.01, ***p < 0.001.

Analysis of colonic tissues revealed that the control mice displayed clear colon mucosa structure, integrated epithelium, and ordered glands with enriched goblet cells, but without obvious inflammatory infiltrates in the lamina propria (Figure 2), whereas the DSS group of mice exhibited severely damaged epithelium with a few epithelial cells, incomplete glands, and wide spreading of inflammatory infiltrates, the hallmarks of inflammatory colonic injury. In contrast, these pathological changes were obviously reduced in the DSS + FMT_h (*p* = 0.0076) and DSS + FMT_m groups of mice. Quantitative analysis indicated that in comparison with that in the control mice, the pathological scores in the colon tissues of the DSS group significantly increased (*p* < 0.0001), which were significantly reduced in both the FMT-treated groups of mice (DSS + FMT_h, *p* = 0.0076; DSS + FMT_m, *p* = 0.0368, Figure 2). ZO-1 and occludin are important tight junction proteins and critical for the integrity of colonic epithelium. Because inflammation can disrupt the integrity of colonic epithelium and the function of intestinal epithelial barrier (Yin et al., 2015), we examined the levels of ZO-1 and occludin expression in colonic tissues of the different groups of mice by IHC. The levels of ZO-1 and occludin expression in the colonic tissues of the DSS group of mice were significantly lower than that of the control mice, but were partially restored in the FMT-treated mice (ZO-1, *p* = 0.0236, *p* = 0.0186; occludin, *p* = 0.0106, *p* = 0.0319).

### FMT Increases the α-Diversity and Changes the Components of Gut Microbiota in Colitis Mice

**FIGURE 3:**
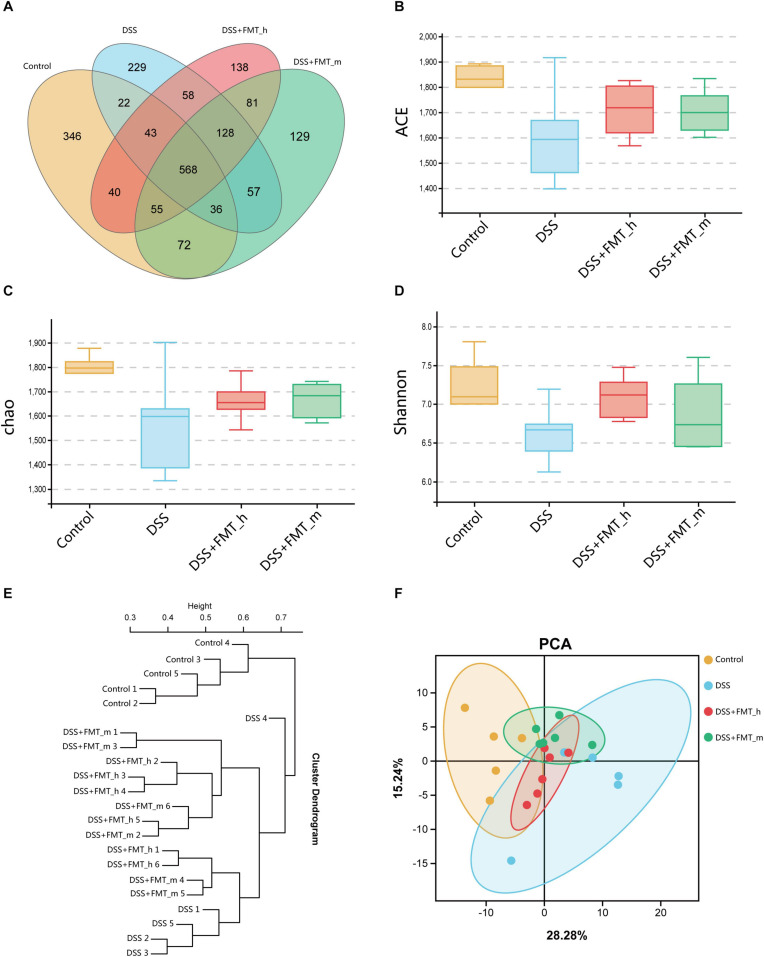
FMT changes the gut microbial diversity in colitis mice. **(A)** Venn diagram displays the common operational taxonomic units (OTUs) and the unique out of the gut microbiota in each group of mice. **(B)** ACE index. **(C)** Chao 1 index. **(D)** Shannon index. **(E)** UPGMA clustering tree exhibits the distribution and relationship of the gut microbiota in each group. Each end branch represents a sample. Generally, samples in the same group were clustered into a large branch, and different groups formed different branches. The vertical axis is the distance scale. Samples in the same branch had similar bacterial community structure. **(F)** Principal Component Analysis (PCA). The PC1 coordinate represents the first principal component, and the percentage in brackets represents the contribution value of the first principal component to the sample difference. The PC2 coordinate represents the second principal component, and the percentage in the bracket represents the contribution value of the second principal component to the sample difference. The colored dots in the panel represent each sample. The more similar the sample composition is, the closer the distance in the PCA diagram is. Samples in different environments indicate their respective aggregation distribution. The α-diversity was determined by species richness (species situation) and species evenness (distribution) using the ACE, Chao 1, and Shannon indexes.

**FIGURE 4:**
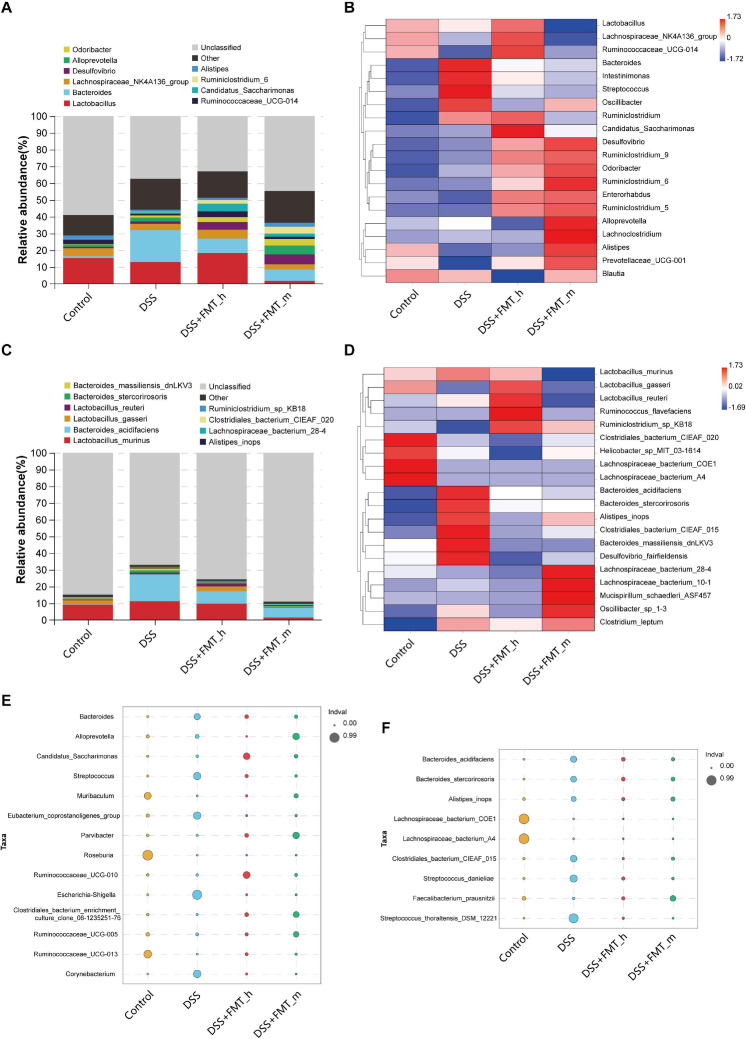
FMT modulates the structure of the gut microbiota in colitis mice. **(A)** Stacked bar plot displays the genus structure of the gut microbiota in each group of mice. **(B)** Heatmap analysis of the difference in genera of the gut microbiota in each group of mice. **(C)** Stacked bar plot of species. **(D)** Heatmap analysis of species. **(E,F)** Bubble diagram analysis of indicators. The bubble size represents the indicator value (IndVal) of the species in the corresponding group, and the bubble color represents the grouping information.

To understand the mechanisms underlying the action of FMT in this model, we collected fecal samples longitudinally, and the distribution of the gut microbiota in the different groups of mice is shown in Figure 3A. Next, we analyzed the α-diversity of the gut microbiota by calculating the ACE, Chao 1, and Shannon indexes. We found that the ACE, Chao 1, and Shannon indexes of the DSS group were markedly lower than those of the control group and they markedly increased in the FMT-treated groups of mice, except for no obviously increased Shannon index in the DSS + FMT___m group (Figures 3B–D). We further analyzed the UPGMA clustering tree and PCA of the gut microbiota in the different groups of mice. We found that the bacterial communities varied among the groups, which was supported by the PCA (Figures 3E,F). The top 10 genera and species of the gut microbiota in the different groups of mice are shown in Figures 4A,C. In comparison with that in the DSS group, increased abundance of *Lactobacillus* (18.30 vs. 12.92%), *Lachnospiraceae_NK4A136_group* (5.39 vs. 3.76%), *Desulfovibrio* (4.48 vs. 1.49%), *Candidatus_Saccharimonas* (4.63 vs. 0.89%), *Ruminococcaceae_UCG-014* (3.43 vs. 0.97%), *Odoribacter* (2.89 vs. 1.30%), *Ruminiclostridium_6* (2.13 vs. 0.33%), and *Alistipes* (1.41 vs. 1.28%) as well as decreased abundance of *Bacteroides* (8.60 vs. 19.00%) and *Alloprevotella* (0.02 vs. 2.29%) were detected in the DSS + FMT_h group. Similarly, increased abundance of *Desulfovibrio* (5.92 vs. 1.49%), *Ruminiclostridium_6* (4.13 vs. 0.33%), *Alloprevotella* (5.28 vs. 2.29%), *Odoribacter* (3.89 vs. 1.30%), *Alistipes* (2.36 vs. 1.28%), *Candidatus_Saccharimonas* (1.85 vs. 0.89%), and *Ruminococcaceae_UCG-014* (1.41 vs. 0.97%), but decreased abundance of *Bacteroides* (6.91 vs. 19.00%), *Lachnospiraceae_NK4A136_group* (3.00 vs. 3.76%), and *Lactobacillus* (1.67 vs. 12.92%) were observed in the DSS + FMT_m group. Furthermore, the abundance of *Lactobacillus_gasseri* (2.90 vs. 0.13%), *Lactobacillus_reuteri* (1.65 vs. 0.87%), and *Ruminiclostridium_sp_KB18* (0.26 vs. 0.05%) increased, whereas the abundance of *Lactobacillus_murinus* (9.70 vs. 11.23%), *Bacteroides_acidifaciens* (7.47 vs. 16.02%), *Bacteroides_stercorirosoris* (0.70 vs. 1.45%), *Bacteroides_massiliensis_dnLKV3* (0.27 vs. 1.12%), *Alistipes_ inops* (0.25 vs. 0.55%), *Clostridiales_bacterium_CIEAF_020* (0.13 vs. 0.17%), and *Lachnospiraceae_bacterium_28-4* (0.03 vs. 0.09%) decreased in the DSS + FMT_h group of mice. Additionally, the abundance of *Lachnospiraceae_bacterium_28-4* (0.54 vs. 0.09%), *Clostridiales_bacterium_CIEAF_020* (0.19 vs. 0.17%), and *Ruminiclostridium_sp_KB18* (0.19 vs. 0.05%) increased, whereas the abundance of *Bacteroides_acidifaciens* (5.76 vs. 16.02%), *Lactobacillus_murinus* (1.47 vs. 11.23%), *Bacteroides_stercorirosoris* (0.73 vs. 1.45%), *Alistipes_inops* (0.41 vs. 0.55%), *Bacteroides_massiliensis_dnLKV3* (0.27 vs. 1.12%), *Lactobacillus_gasseri* (0.06 vs. 0.13%), and *Lactobacillus_reuteri* (0.03 vs. 0.87%) decreased in the DSS + FMT_m group of mice. The difference in the abundance of genera and species with > 0.1% of the number of species tags in the sample/the total number of tags in the samples is illustrated by the heatmaps (Figures 4B,D). Apparently, increased abundance of *Candidatus_Saccharimonas*, *Desulfovibrio*, *Ruminiclostridium_9*, *Odoribacter*, *Ruminiclostridium_6*, *Enterorhabdus*, and *Ruminiclostridium_5*, but decreased abundance of *Bacteroides*, *Intestinimonas*, *Streptococcus*, and *Oscillibacter* were detected in the FMT-treated mice, relative to that in the DSS group. Compared with that in the DSS + FMT_m group, the abundance of *Lactobacillus*, *Lachnospiraceae_NK4A136_group*, and *Ruminococcaceae_UCG-014* increased, but the abundance of *Alloprevotella*, *Lachnoclostridium*, *Alistipes*, and *Prevotellaceae_UCG-001* decreased in the DSS + FMT_h group of mice. At the species level, the abundance of *Bacteroides_acidifaciens*, *Bacteroides_stercorirosoris*, *Alistipes_inops*, *Clostridiales_ bacterium_CIEAF_015*, *Bacteroides_massiliensis_dnLKV3*, and *Desulfovibrio_fairfieldensis* decreased, whereas the abundance of *Ruminiclostridium_sp_KB18* increased in the FMT-treated mice. Moreover, the abundance of *Lactobacillus_gasseri*, *Lactobacillus_reuteri*, and *Ruminococcus_flavefaciens* increased, whereas the abundance of *Lachnospiraceae_bacterium_28-4*, *Lachnospiraceae_bacterium_10-1*, *Mucispirillum_schaedleri_ ASF457*, and *Oscillibacter_sp_1-3* decreased in the DSS + FMT_h group, relative to that in the DSS + FMT_m group of mice.

Based on the occurrence frequency and abundance of species among the groups, we analyzed the indicator species by the indicator analysis and calculated the indicator value (IndVal) of each species in each group (bubble charts, Figures 4E,F). In comparison with that in the control group, the abundance of *Escherichia–Shigella*, *Corynebacterium*, *Eubacterium_coprostanoligenes_group*, *Streptococcus*, and *Bacteroides* significantly increased, whereas the abundance of *Roseburia*, *Ruminococcaceae_UCG-013*, and *Muribaculum* significantly reduced in the colitis mice (Figure 4E). The abundance of *Muribaculum*, *Ruminococcaceae_UCG-013*, *Clostridiales_bacterium_enrichment_culture_clone_06-1235251 -76*, *Parvibacter*, *Candidatus_Saccharimonas*, and *Rumino coccaceae_UCG-010* significantly increased, whereas the abundance of *Bacteroides*, *Streptococcus*, *Corynebacterium*, *Eubacterium_coprostanoligenes_group*, *Escherichia–Shigella*, and *Alloprevotella* significantly decreased in the DSS + FMT_h group, relative to that in the DSS + FMT_m group. Similarly, the abundance of *Roseburia*, *Ruminococcaceae_UCG-013*, *Ruminococcaceae_UCG-010*, *Ruminococcaceae_UCG-005*, *Clostridiales_bacterium_enrichment_culture_clone_06-1235251-76*, and *Parvibacter* was significantly higher than that of other groups. At the species level, decreased abundance of *Streptococcus_thoraltensis_DSM_12221*, *Streptococcus_danieliae*, *Clostridiales_bacterium_CIEAF_015*, *Bacteroides_acidifaciens*, *Bacteroides_stercorirosoris*, and *Alistipes_inops*, but increased abundance of *Faecalibacterium_prausnitzii*, *Lachnospiraceae_bacterium_COE1*, and *Lachnospiraceae_ bacterium_A4* were detected in colitis mice (Figure 4F). However, the abundance of *Streptococcus*_ *thoraltensis_DSM_12221*, *Streptococcus_danieliae*, *Clostridiales_ bacterium_CIEAF_015*, *Bacteroides_acidifaciens*, *Bacteroides_stercorirosoris*, and *Alistipes_inops* were significantly lower in both the DSS + FMT_h and DSS + FMT_m groups, while the abundance of *Faecalibacterium_prausnitzii* were significantly higher than that in the DSS group of mice.

### FMT Restores Fecal Butyric Acid Levels in Colitis Mice

**FIGURE 5:**
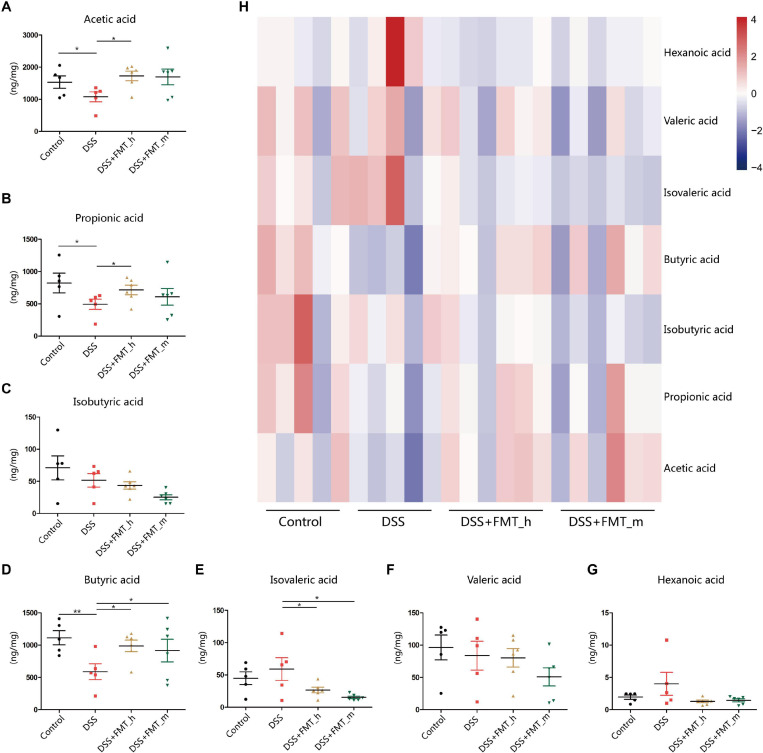
FMT restores some types of fecal short-chain fatty acid (SCFA) levels in colitis mice. **(A–G)** The contents of fecal SCFA types in each group of mice. **(H)** Heatmap analysis of the contents of different types of fecal SCFAs in mice. *p < 0.05, **p < 0.01.

Metabonomics analysis of SCFAs indicated that the levels of fecal acetic acid, propionic acid, and butyric acid in the DSS group were significantly lower than that in the control mice (*p* = 0.0453, *p* = 0.0466, and *p* = 0.0063, respectively, Figures 5A,B,D). Furthermore, compared with that in the DSS group, the levels of fecal acetic, propionic, and butyric acids were partially or completely restored in the DSS + FMT_h group of mice (*p* = 0.0073, *p* = 0.0361, and *p* = 0.0126, respectively), but only fecal butyric acid levels were significantly restored in the DSS + FMT_m group of mice (*p* = 0.0473, Figure 5D). In addition, DSS-induced colitis did not significantly change the levels of fecal isobutyric, isovaleric, valeric, and hexanoic acids in mice (Figures 5C,E–G), but transplantation with the gut microbiota from human or mice significantly decreased the levels of fecal isovaleric acid in colitis mice (Figure 5F). Levels of different type of fecal SCFAs in mice were illustrated by the heatmap (Figure 5H). Such data suggest that fecal butyric acid level may be a potential marker for evaluating the efficacy of FMT for UC.

### Butyric Acid Increases the α-Diversity and Changes the Gut Microbiota Structure in DSS-Induced Colitis Mice

**FIGURE 6:**
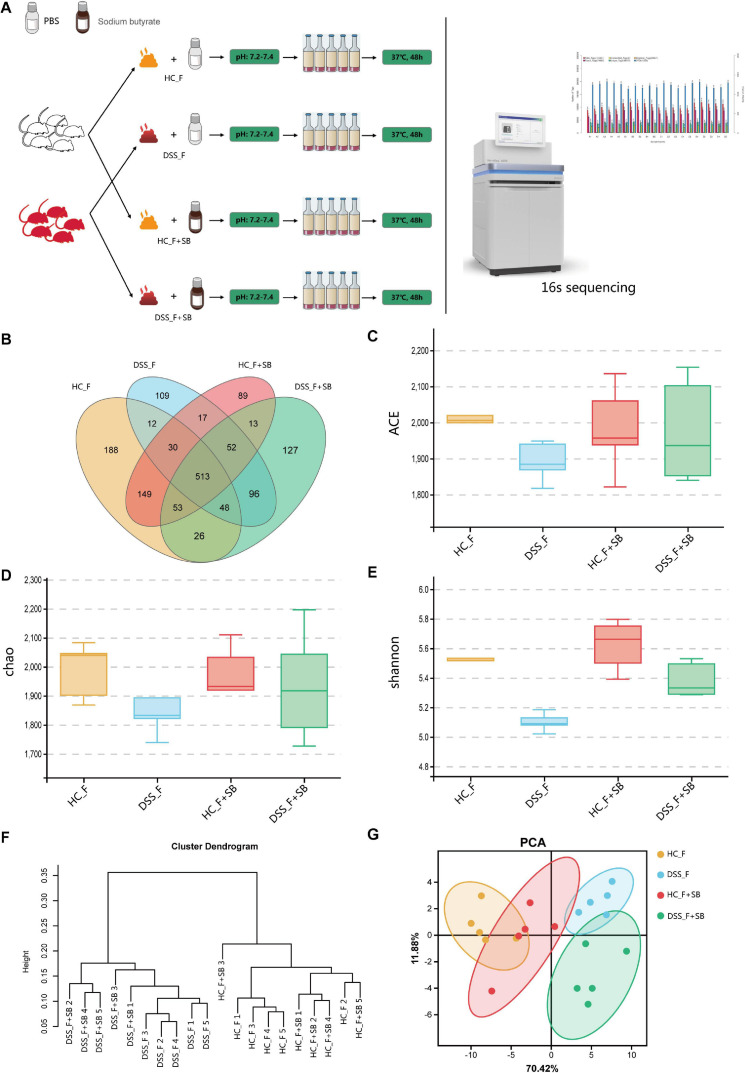
Anaerobic culture with butyrate increases fecal microbial diversity *in vitro*. **(A)** Schematic diagram of the experimental design. The fecal samples were collected from healthy and colitis mice in an anaerobic condition and cultured in the specific media (pH 7.2–7.4) in the presence of sodium butyrate in an anaerobic condition for 48 h. The microbiota were analyzed by 16S sequencing. **(B)** Venn diagram. **(C)** ACE index. **(D)** Chao 1 index. **(E)** Shannon index. **(F)** UPGMA clustering tree. **(G)** PCA. HC_F: fecal samples from healthy mice were cultured without sodium butyrate; DSS_F: fecal samples from colitis mice were cultured without sodium butyrate; HC_F + SB: fecal samples from healthy mice were cultured with sodium butyrate; DSS_F + SB: fecal samples from colitis mice were cultured with sodium butyrate.

Butyric acid is one of the most important metabolites in the gut microbiota, and its decrease is associated with the development of inflammatory bowel disease (IBD) in humans (Parada Venegas et al., 2019). Previous studies have found that butyric acid can act as an intestinal epithelial energy substance, a deacetylase inhibitor, an activator of some G protein coupled receptors, and others to improve intestinal inflammation (Zhuang et al., 2019; Zhou et al., 2020). Next, we tested whether the butyric acid increase could be the outcome of an improvement in intestinal inflammation or an alteration in the gut microbiota. First, we tested whether butyric acid could modulate the diversity and components of the gut microbiota in colitis mice. We isolated the fecal samples from the different groups of mice under an anaerobic working condition and mixed an equal amount of fecal samples with the same volume of PBS or sodium butyrate, followed by anaerobically cultured them in media for 48 h (Figure 6A). Subsequently, we analyzed the abundance and components of microbiota by 16S sequencing. The results revealed that in comparison with that in the HC_F group, the structure of microbial community in the DSS_F group was altered with markedly lower FM diversity. Culture with sodium butyrate significantly increased the diversity of microbiota in the HC_F + SB and DSS_F + SB groups, compared with the HC_F and DSS_F groups, respectively (Figures 6B–E). In addition, culture with sodium butyrate significantly increased the α-diversity of microbiota in the DSS_F + SB group, relative to that in the DSS_F group (Figures 6F,G).

**FIGURE 7:**
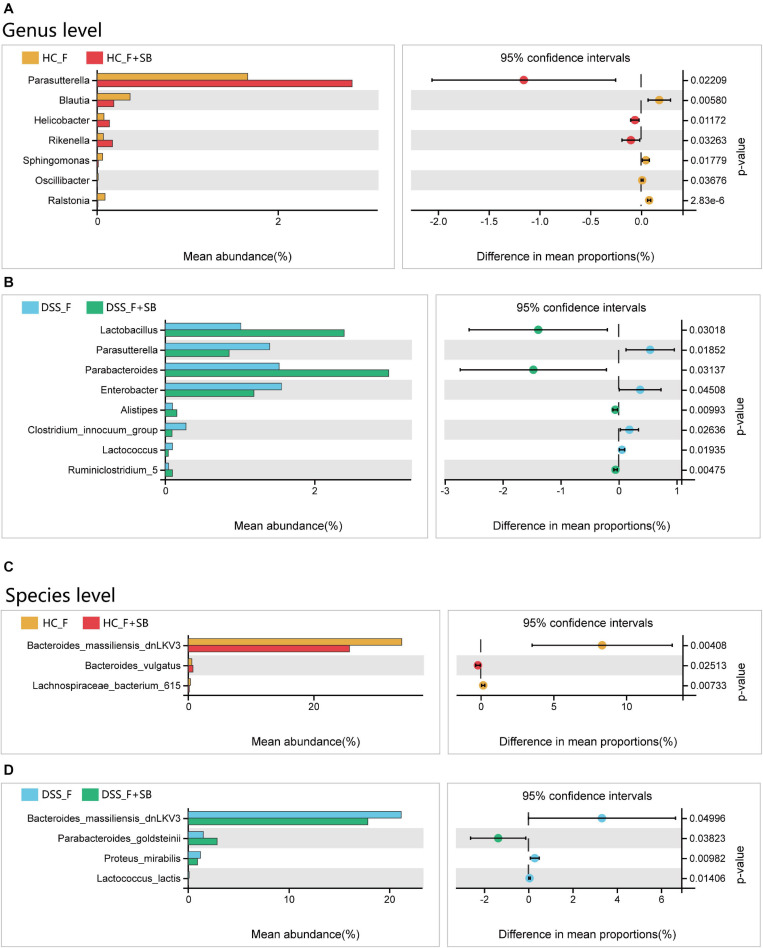
Butyrate modulates the structure of fecal microbiota *in vitro*. **(A,B)** Butyrate alters the structure of fecal microbiota from healthy control mice at the genus and species levels, respectively. **(C,D)** Butyrate changes the structure of fecal microbiota from colitis mice at the genus and species levels, respectively.

At the genus level, butyrate significantly increased the abundance of *Parasutterella*, *Helicobacter*, and *Rikenella*, but decreased that of *Blautia*, *Sphingomonas*, *Oscillibacter*, and *Ralstonia* in the HC_F group (Figure 7A). Furthermore, butyrate significantly increased the abundance of *Lactobacillus*, *Parabacteroides*, *Alistipes*, and *Ruminiclostridium_5*, but decreased that of *Parasutterella*, *Enterobacter*, *Clostridium_innocuum_group*, and *Lactococcus* in the DSS_F + SB group (Figure 7B). At the species level, butyrate significantly increased the abundance of *Bacteroides_vulgatus*, but decreased that of *Bacteroides_massiliensis_dnLKV3* and *Lachnospiraceae_bacterium_615* in the HC-F + SB group, compared with that in the HC-F group (Figure 7C). In addition, butyrate significantly enlarged the population of *Parabacteroides_goldsteinii*, but limited that of *Bacteroides_massiliensis_dnLKV3*, *Proteus_mirabilis*, and *Lactococcus_lactis* in the DSS_F + SB group, relative to that in the DSS_F group (Figure 7D).

### Addition of *C. butyricum*-Containing Probiotics Enhances the Therapeutic Efficacy of FMT for UC Patients at 3 Months Post-FMT

Oral probiotics, containing abundant *C. butyricum TO-A*, *B. mesentericus TO-A*, and *S. faecalis T-110*, “Shiyi” from Toa Pharmaceutical (Japan), have been used as an addition of other therapies for UC patients. We retrospectively analyzed the efficacy of FMT alone or combined with Shiyi on clinical measures in 45 UC patients from 2017 to 2019 (Supplementary Table 4). We found that the addition of Shiyi to the standard therapies in 11 out of 45 UC patients did not significantly enhance the therapeutic responses at 1 month post-FMT, but significantly enhanced the therapeutic efficacy of FMT by increasing the therapeutic response rate (9/11 vs. 15/34 in the FMT alone group) at 3 months post-FMT (χ^2^ = 3.9477, *p* = 0.0469). Therefore, the addition of oral *C. butyricum*-containing probiotics enhances the therapeutic efficacy of FMT for UC patients at 3 months post-FMT.

## Discussion

UC is one of the common forms of IBD (Kirsner, 1988) and is associated with dysfunctional immune responses, altered gut microbiota, genetic susceptibility, and environmental factors. Although the FMT has reported adverse effects in a few cases (Marcella et al., 2021), its therapeutic efficacy for inducing a short period of remission of active UC, especially for those with mild to moderate UC, has been widely recognized. However, how modification of FMT therapy to induce long-term remission in UC patients has not been clarified.

In this study, we first analyzed the gut microbiota in 12 fecal samples from UC patients receiving FMT because those patients did not receive any antibiotics, probiotics, hormones, and other drugs for at least 3 months pre-FMT and post-FMT, and these drugs can significantly change the structure of the gut microbiota. We found the presence of corresponding donor FMT suspension and high abundance of SCFA-producing bacteria (especially for butyrate-producing bacteria) in fecal samples from those patients, accompanied by higher levels of fecal SCFAs in some patients. Such data suggest that higher levels of fecal SCFAs, particularly for butyric acid, may be associated with the therapeutic efficacy of FMT in UC patients. Next, we found that compared with the untreated colitis mice, FMT with the gut microbiota from the same healthy donor as for human UC patients or from healthy mice significantly reduced the DAI scores, colonic pathological changes, and serum inflammatory IL-6 and TNF-α levels, but increased colon lengths and the levels of tight junction protein expression in colitis mice. There was no significant difference in the therapeutic efficacy between the mice receiving human and mouse sources of the gut microbiota. Such data demonstrated that the FMT significantly ameliorated colonic inflammation and damages in colitis mice, regardless of human or mouse source of the gut microbiota.

To understand the therapeutic action of FMT, we analyzed the gut microbiota from the different groups of mice. Compared with untreated colitis mice, FMT remarkably increased the α-diversity of the gut microbiota and changed the structure of the gut microbiota in colitis mice. Particularly, FMT significantly increased the abundance of the top 10 species, including *Desulfovibrio*, *Odoribacter*, *Ruminococcaceae_UCG-014*, *Candidatus_Saccharimonas*, *Ruminiclostridium_6*, and *Alistipes*, but decreased *Bacteroides* in colitis mice. Interestingly, FMT from human donors significantly increased the abundance of *Lactobacillus* and *Lachnospiraceae_NK4A136_group*, but decreased *Alloprevotella* in colitis mice, relative to those receiving FMT from mouse. Furthermore, FMT significantly enlarged the numbers of *Ruminiclostridium_sp_KB18*, but reduced *Lactobacillus_murinus*, *Bacteroides_acidifaciens*, *Bacteroides_stercorirosoris*, *Bacteroides_massiliensis_dnLKV3*, and *Alistipes_inops* in colitis mice. FMT from human donor increased the abundance of *Lactobacillus* genus, including *Lactobacillus_gasseri* and *Lactobacillus_reuteri* species, but decreased *Lachnospiraceae_bacterium_28-4* and *Clostridiales_bacterium_CIEAF_020* in colitis mice, whereas FMT from mice had the opposite effects on these bacteria in the gut microbiota of colitis mice. It was notable that FMT also significantly increased the abundance of *Muribaculum*, *Ruminococcaceae_UCG-013*, *Clostridiales_bacterium_enrichment_culture_clone_06-1235251- 76*, *Parvibacter*, *Candidatus_Saccharimonas*, and *Rumi nococcaceae_UCG-010*, but decreased *Bacteroides*, *Streptococcus*, *Corynebacterium*, *Eubacterium_coprostanoligenes_group*, *Escherichia–Shigella*, and *Alloprevotella* in the gut microbiota of colitis mice. FMT from mice enlarged the numbers of *Roseburia*, *Ruminococcaceae_UCG-013*, *Ruminococcaceae_UCG-010*, *Ruminococcaceae_UCG-005*, *Clostridiales_bacterium_enrichment_culture_clone_06-1235251-76*, and *Parvibacter*; but minimized *Bacteroides*, *Streptococcus*, *Eubacterium_coprostanoligenes_group*, *Escherichia–Shigella*, and *Corynebacterium* in colitis mice. At the species level, the FMT significantly reduced the abundance of *Streptococcus_thoraltensis_DSM_12221*, *Streptococcus_danieliae*, *Clostridiales_bacterium_CIEAF_015*, *Bacteroides_acidifaciens*, *Bacteroides_stercorirosoris*, and *Alistipes_inops*, but expanded the population of *Faecalibacterium_prausnitzii* in colitis mice. Therefore, FMT increased the diversity of the gut microbiota and changed the structure of the gut microbiota in colitis mice by increasing the abundance of *Ruminococcaceae*, *Roseburia*, *Parvibacter*, *Candidatus_Saccharimonas*, and *Faecalibacterium_prausnitzii* and decreasing *Bacteroides*, *Streptococcus*, *Eubacterium_coprostanoligenes_group*, *Escherichia–Shigella*, and *Corynebacterium*. Remarkably, the increased abundant *Ruminococcaceae*, *Roseburia*, and *Faecalibacterium_prausnitzii* are important butyrate-producing bacteria, whereas those with decreased abundance of bacteria are associated with intestinal inflammation (*Streptococcus_thoraltensis*, *Streptococcus_danieliae*, *Bacteroides_acidifaciens*, *Escherichia–Shigella*, *Corynebacterium*). Such data suggest that FMT modulated the abundance of components in the gut microbiota, limiting colonic inflammation, contributing to its therapeutic efficacy in colitis mice.

Consistent with the increased abundance of butyrate-producing bacteria in the gut microbiota following FMT in mice, we found that FMT significantly increased fecal butyric acid contents and FMT from human elevated the levels of fecal acetic and propionic acids in colitis mice. More importantly, we found that anaerobic culture of fecal samples from colitis and healthy mice with butyrate significantly enhanced the diversity of microbiota and changed their structure *in vitro* by increasing the abundance of *Parasutterella*, *Helicobacter*, and *Rikenella* as well as *Bacteroides_vulgatus*, but decreasing *Blautia*, *Sphingomonas*, *Oscillibacter*, and *Ralstonia*, particularly for *Bacteroides_massiliensis_dnLKV3* and *Lachnospiraceae_bacterium_615* in microbiota from healthy mice. Similarly, butyrate significantly increased the abundance of *Lactobacillus*, *Parabacteroides*, *Alistipes*, and *Ruminiclostridium_5* as well as *Parabacteroides_goldsteinii*, but decreased *Parasutterella*, *Enterobacter*, *Clostridium_innocuum_group*, and *Lactococcus* as well as *Bacteroides_massiliensis_dnLKV3*, *Proteus_mirabilis*, and *Lactococcus_lactis* in microbiota from colitis mice. Moreover, retrospective analysis of UC patients with FMT alone or combination of FMT and *C. butyricum*-enriched probiotics revealed that the addition of *C. butyricum*-enriched probiotics significantly prolonged the therapeutic effect of FMT in UC patients by significantly increasing the response rate at 3 months post-FMT. Collectively, these data suggest that the combination of FMT with butyric acid-related reagents may prolong the remission period of FMT in UC patients. The abundance of SCFA-producing bacteria and the contents of fecal butyric acid in the recipients may be valuable biomarkers for evaluating the therapeutic efficacy of FMT in UC patients. We experienced that it was important to exclude those fecal samples from individuals with antibiotics, probiotics, hormones, and other drugs for gut microbiota research. Second, it was critical to collect fecal samples and transfer them to –80°C as soon as possible for the metabonomic analysis to avoid the influence of bacterial metabolism and metabolites. Furthermore, we used a special anaerobic workstation to isolate fecal samples from mice to cultivate the FM in a special culture medium, which was necessary for expending the majority of the gut microbiota *in vitro*. Furthermore, our findings highlight that (1) fecal butyric acid may be a potential biomarker for evaluating the efficacy of FMT in patients with recurrent UC after FMT treatment; (2) there is an “interaction” between gut microbiota and their metabolites; (3) the modified method is an extension of culturing omics, which provides a reference for the study of the gut microbiota; and (4) FMT combination with *C. butyricum*-containing probiotics significantly prolonged the therapeutic efficacy of FMT in UC remission in the clinic.

However, we recognized that our study had limitations. First, the sample size for 16S sequencing (*n* = 12) and targeted SCFAs metabonomic analysis (*n* = 5) was small due to restriction of the recipients without any drug treatment that had potential effect on the gut microbiota. All 12 UC patients received FM from the same healthy donor, which might have a donor bias. In future studies, we should recruit more healthy donors for FMT to avoid the bias of a single donor. In addition, we should consider the gender, age, and other factors of donors because these factors may affect the efficacy of FMT for UC patients (Fang et al., 2021). Second, the conditions for *in vitro* culture of FM remained to be further optimized. We did not test other SCFAs. Given that increased levels of fecal acetic acid were detected in UC patients after FMT and the levels of fecal acetic acid were significantly associated inversely with the severity of UC in patients, we should examine the potential role of other SCAFs in the therapeutic effect of FMT. Furthermore, we prepared FM for FMT by washing them three times, which should be considered as washed microbiota transplantation (WMT) (Zhang and Liu, 2020). This process should remove the majority of bacterial metabolites. Actually, we failed to detect SCFAs in samples from the same healthy donor’s FMT suspension. Moreover, we only treated UC patients with 2–3 FMT and monitored patients at 3 months post-FMT. Given that recent studies have shown that frequent FMT and continual monitoring of patients for 6 months are feasible and valuable (Paramsothy et al., 2019; Mañkowska-Wierzbicka et al., 2020), we should consider more frequent FMT for UC patients and extend our longitudinal studies for a longer period. Finally, we did not analyze colonic tissues histologically, intestinal barrier function, and inflammatory measures in those UC patients, which might miss important information on the action of FMT in UC patients. Therefore, further studies with bigger samples, particularly from those receiving FMT with fresh and unwashed FM from multiple healthy donors, are warranted to analyze the microbial structure and fecal metabolites of matched healthy donors. Potentially, we may apply the strategy of selective FM (Zhang et al., 2020) by isolating and culturing functional bacteria for FMT in UC patients.

## Conclusion

Our data indicated that FMT increased the diversity of the gut microbiota in UC patients and FMT, from either healthy donors or mice, also significantly changed the gut microbiota with unique characteristics in colitis mice, accompanied by ameliorating clinical symptoms. Particularly, FMT increased the abundance of butyrate-producing bacteria in the gut microbiota and the contents of fecal SCFAs, such as butyric acid, in the recipients. Furthermore, anaerobic culture of microbiota from healthy donors or mice with butyrate significantly changed the structure of microbiota *in vitro*. Such exploratory protocol may be used as a reference for future gut microbiota research. Finally, retrospective analysis revealed that the combination of FMT with *C. butyricum*-enriched probiotics significantly prolonged the remission of UC patients. Our findings suggest that fecal butyric acid level may be a biomarker for evaluating the efficacy of FMT for UC and addition of butyrate-producing bacteria may prolong the therapeutic effect of FMT on UC by changing the gut microbiota.

## Data Availability Statement

The datasets presented in this study can be found in online repositories. The names of the repository/repositories and accession number(s) can be found below: The original contributions presented in the study are publicly available. These data can be found here: http://www.ncbi.nlm.nih.gov/bioproject/684887, BioProject ID is PRJNA684887; http://www.ncbi.nlm.nih.gov/bioproject/684888, BioProject ID is PRJNA684888.

## Ethics Statement

Use of stool samples was approved by the Biological Sample Bank of Guangzhou First People’s Hospital. The patients/participants provided their written informed consent to participate in this study. The animal study was reviewed and approved by the Medical Ethics Committee of Guangzhou First People’s Hospital. Written informed consent was obtained from the individual(s) for the publication of any potentially identifiable images or data included in this article.

## Author Contributions

H-MX designed the study and drafted the manuscript. H-LH and JX performed the animal experiments, statistical analysis, and interpretation of the data. JH, CZ, and YP participated in the animal experiments, recorded the general status of experimental animals, and collected the samples and related tests. H-LZ, W-QH, C-YC, and Y-JZ interpreted the data and revised the manuscript. Y-LZ and Y-QN designed and organized the study, interpreted the data, and revised the manuscript. All authors contributed to the article and approved the submitted version.

## Conflict of Interest

The authors declare that the research was conducted in the absence of any commercial or financial relationships that could be construed as a potential conflict of interest.

## Footnotes

1 http://cran.r-project.Org

2 http://CRAN.R-project.org/package=ggplot2; version 2.2.1
