# Supplementary material for: Cross-Talk Between Butyric Acid and Gut Microbiota in Ulcerative Colitis Following Fecal Microbiota Transplantation
Source: Front Microbiol. 2021 Apr 12;12:658292. doi: 10.3389/fmicb.2021.658292 (PMC8071877; doi:10.3389/fmicb.2021.658292)
Supplement: Supplementary file 1 [file Table_1.DOCX]

| **Supplementary Table 1. The demographic and clinical characteristics of 12 UC patients treated with FMT** | | | | | | |
| --- | --- | --- | --- | --- | --- | --- |
| Patient ID | Sex | Age (years) | Disease duration(months) | Montreal type | Total Mayo scores | FMT times |
| 1 | M | 33 | 27 | E2 | 8 | 2 |
| 2 | M | 33 | 4 | E2 | 5 | 3 |
| 3 | F | 41 | 36 | E2 | 8 | 2 |
| 4 | M | 54 | 60 | E2 | 6 | 2 |
| 5 | F | 43 | 6 | E3 | 6 | 2 |
| 6 | F | 62 | 12 | E3 | 6 | 2 |
| 7 | F | 20 | 48 | E3 | 9 | 3 |
| 8 | F | 49 | 72 | E1 | 6 | 2 |
| 9 | M | 36 | 120 | E1 | 6 | 2 |
| 10 | M | 36 | 72 | E2 | 5 | 2 |
| 11 | F | 60 | 180 | E3 | 8 | 2 |
| 12 | M | 25 | 30 | E3 | 9 | 3 |

Notes: The fecal microbiota were prepared from a male healthy donor at 29 years of age and transplanted into individual patients through colonic TET. UC: ulcerative colitis; FMT: fecal microbiota transplantation; TET: transendoscopic enteral tubing

| **Supplementary Table 2. Evaluation of clinical symptoms in 12 UC patients before and after FMT** | | | | | | | | | |
| --- | --- | --- | --- | --- | --- | --- | --- | --- | --- |
| Patient ID | Follow-up  (mo) | Defecation  (/d) | Gross blood stool  (/d) | FOBT | CRP  (mg/L) | PCT  (ng/ml) | ESR  (mm/h) | Partial Mayo scores | Responses  (Yes/No) |
| 1 | 0 | 8 | 6 | (+) | 2.05 | 0.026 | 7 | 6 | No |
|  | 1 | 4 | 4 | (+) | / | / | / | 5 |  |
|  | 3 | 6 | 6 | (+) | / | / | / | 6 |  |
| 2 | 0 | 7 | 2 | (+) | 1.25 | 0.072 | 5 | 4 | No |
|  | 1 | 6 | 3 | (+) | 0.364 | 0.029 | / | 4 |  |
|  | 3 | 6 | 2 | (+) | / | / | / | 4 |  |
| 3 | 0 | 10 | 6 | (+) | 0.6 | 0.026 | 10 | 6 | Yes |
|  | 1 | 4 | 3 | (+) | 1.17 | <0.02 | 20 | 3 |  |
|  | 3 | 5 | 1 | (+) | 4.31 | <0.05 | 14 | 4 |  |
| 4 | 0 | 5 | 3 | (+) | 0.9 | / | 5 | 4 | No |
|  | 1 | 6 | 2 | (+) | 1.63 | / | 5 | 4 |  |
|  | 3 | 6 | 3 | (+) | 1.25 | / | 3 | 5 |  |
| 5 | 0 | 5 | 3 | (+) | 6.32 | 0.78 | 53 | 4 | No |
|  | 1 | 6 | 2 | (+) | 7.23 | / | 49 | 4 |  |
|  | 3 | 4 | 3 | (+) | 7.85 | / | 56 | 4 |  |
| 6 | 0 | 6 | 4 | (+) | 0.75 | 0.056 | 5 | 4 | Yes |
|  | 1 | 3 | 0 | (-) | 0.441 | <0.02 | 3 | 1 |  |
|  | 3 | 6 | 4 | (+) | 0.22 | <0.02 | 3 | 4 |  |
| 7 | 0 | >10 | >6 | (+) | 20.5 | 0.035 | 15 | 7 | Yes |
|  | 1 | 5 | 2 | (+) | 0.813 | 0.032 | 10 | 3 |  |
|  | 3 | 5 | 4 | (+) | 1.85 | 0.75 | 15 | 5 |  |
| 8 | 0 | 3 | 3 | (+) | 1.08 | <0.02 | 27 | 4 | Yes |
|  | 1 | 1 | 0 | (-) | 0.274 | <0.02 | 15 | 1 |  |
|  | 3 | 3 | 2 | (+) | 0.457 | 0.026 | 25 | 3 |  |
| 9 | 0 | 5 | 3 | (+) | <0.156 | <0.02 | 3 | 4 | Yes |
|  | 1 | 2 | 0 | (-) | 0.172 | <0.02 | 1 | 1 |  |
|  | 3 | 4 | 2 | (+) | 0.329 | <0.02 | 3 | 4 |  |
| 10 | 0 | 4 | 2 | (+) | 0.75 | 0.022 | 6 | 4 | Yes |
|  | 1 | 1 | 0 | (-) | 0.811 | 0.032 | 8 | 1 |  |
|  | 3 | 3 | 2 | (+) | 0.854 | <0.02 | 5 | 3 |  |
| 11 | 0 | 5 | 3 | (+) | 0.432 | <0.02 | 5 | 5 | Yes |
|  | 1 | 2 | 0 | (+) | 0.269 | <0.02 | 5 | 2 |  |
|  | 3 | 3 | 1 | (+) | 0.28 | <0.02 | 3 | 3 |  |
| 12 | 0 | 5 | 3 | (+) | 0.22 | 0.064 | 3 | 6 | Yes |
|  | 1 | 2 | 0 | (-) | <0.162 | 0.037 | 2 | 2 |  |
|  | 3 | 4 | 2 | (+) | 0.396 | 0.046 | 6 | 5 |  |
| UC: ulcerative colitis; FMT: fecal microbiota transplantation; FOBT: fecal occult blood test; CRP: C-reactive protein; PCT: procalcitonin; ESR: erythrocyte sedimentation rate. | | | | | | | | | |

| **Supplementary Table 3. Average relative abundance of taxonomy in each group at genus level** | | | | |
| --- | --- | --- | --- | --- |
| **Taxonomy** | **Pre-FMT** | **Post-FMT (1 mo)** | **Post-FMT (3 mo)** | **Donor** |
| *Parvimonas* | 0.0000908936 | 0.000316146 | 0.000221301 | 0.0000395112 |
| *Lachnospira* | 0.0000592906 | 0.000624404 | 0.00003162 | 0.002122152 |
| *Faecalibacterium* | 0.007291193 | 0.051488866 | 0.006334842 | 0.045924638 |
| *Ruminococcus_1* | 0.0000079 | 0.0000513786 | 0.00003162 | 0.014855065 |
| *Pediococcus* | 0.014700947 | 0.000051363 | 0.00004346 | 0 |
| *Roseburia* | 0.002410647 | 0.001011686 | 0.0000830054 | 0.009002351 |
| *Akkermansia* | 0.00020549 | 0.023790235 | 0.000162019 | 0.037175206 |
| *Ruminococcaceae_UCG-002* | 0.00056907 | 0.007484835 | 0.00003952 | 0.019889743 |
| *Bilophila* | 0.094809224 | 0.007065937 | 0.001315969 | 0.003604102 |
| *Methanobrevibacter* | 0.0000316 | 0.011776562 | 0.00003162 | 0.014558676 |
| *Faecalitalea* | 0.036131926 | 0.001430576 | 0.000395191 | 0.000106713 |
| *Lachnospiraceae_NK4A136_group* | 0.000106705 | 0.00159655 | 0.0000829942 | 0.018854354 |
| *Barnesiella* | 0.00411785 | 0.00300737 | 0.000264799 | 0.018067932 |
| *Erysipelotrichaceae_UCG-003* | 0.000264771 | 0.0050821 | 0.068007671 | 0.000509791 |
| *Collinsella* | 0.012349582 | 0.060337094 | 0.010385513 | 0.005224367 |
| *Campylobacter* | 0.0000079 | 0.00000396 | 0.000055323 | 0.000379372 |
| *Peptostreptococcus* | 0.00001976 | 0.007089648 | 0 | 0.00001582 |
| *[Ruminococcus]_gnavus_group* | 0.014017262 | 0.000624403 | 0.029453259 | 0.000071123 |
| *Alistipes* | 0.001327828 | 0.010480349 | 0.000569081 | 0.020107096 |
| *Phascolarctobacterium* | 0.029757553 | 0.000252916 | 0.000604636 | 0.007409749 |
| *Clostridium_sensu_stricto_1* | 0.000383327 | 0.015380663 | 0.000727143 | 0.010942718 |
| *Gemella* | 0.004967492 | 0.02558043 | 0.008298924 | 0.000225256 |
| *Peptoniphilus* | 0.0000948374 | 0.011021768 | 0.00079037 | 0.00000396 |
| *Bifidobacterium* | 0.062356498 | 0.071165208 | 0.131122923 | 0.002971803 |
| *Veillonella* | 0.001711166 | 0.000205505 | 0.001505661 | 0.002331601 |
| *Streptococcus* | 0.012815902 | 0.007887925 | 0.22903041 | 0.011642198 |
| *Anaerostipes* | 0.000671814 | 0.000829892 | 0.002110301 | 0.008516272 |
| *Catenibacterium* | 0.000339857 | 0.113019423 | 0.000331957 | 0 |
| *Ruminococcaceae_NK4A214_group* | 0.00002372 | 0.004501168 | 0.0000632268 | 0.020383726 |
| *[Ruminococcus]_torques_group* | 0.00038334 | 0.002390883 | 0.0000434912 | 0.010618665 |
| *Lachnoclostridium* | 0.011788417 | 0.000928689 | 0.000371461 | 0.005125571 |
| *Ruminococcaceae_UCG-014* | 0.0000908936 | 0.00443005 | 0.000142264 | 0.022624434 |
| *Ruminococcus_2* | 0.00002768 | 0.001379206 | 0.00001976 | 0.015661246 |
| *[Eubacterium]_coprostanoligenes_group* | 0.000553261 | 0.034491889 | 0.000225249 | 0.131818452 |
| *Aeromonas* | 0.07678081 | 0.00001976 | 9.88092E-05 | 0.0000829712 |

FMT: fecal microbiota transplantation; Pre-FMT: patients before receiving FMT; Post-FMT (1 mo): patients who received FMT treatment post 1 month, Post-FMT (3 mo): patients who received FMT treatment post 3 month, Donor: healthy donor.

| **Supplementary Table 4. The demographic and clinical characteristics of 45 UC patients for retrospective study** | | | | | | | | | | | |
| --- | --- | --- | --- | --- | --- | --- | --- | --- | --- | --- | --- |
| **Patient ID** | **Sex** | **Age (year)** | **Disease duration (mo)** | **Montreal type** | **Total Mayo scores** | **FMT times** | **FMT route** | **Donor ID** | **Responses**  **(Yes/No)** | **Effect for 3 mo**  **(Yes/No)** | **Use of probiotics containing**  ***Clostridium butyricum*** |
| 1 | M | 33 | 27 | E2 | 8 | 2 | Colonic TET | 1 | No | No | / |
| 2 | M | 33 | 4 | E2 | 5 | 3 | Colonic TET | 1 | No | No | / |
| 3 | F | 41 | 36 | E2 | 8 | 2 | Colonic TET | 1 | Yes | Yes | / |
| 4 | M | 54 | 60 | E2 | 6 | 2 | Colonic TET | 1 | No | No | / |
| 5 | F | 43 | 6 | E3 | 6 | 2 | Colonic TET | 1 | No | No | / |
| 6 | F | 62 | 12 | E3 | 6 | 2 | Colonic TET | 1 | Yes | Yes | / |
| 7 | F | 20 | 48 | E3 | 9 | 3 | Colonic TET | 1 | Yes | Yes | / |
| 8 | F | 49 | 72 | E1 | 6 | 2 | Colonic TET | 1 | Yes | Yes | / |
| 9 | M | 36 | 120 | E1 | 6 | 2 | Colonic TET | 1 | Yes | No | / |
| 10 | M | 36 | 72 | E2 | 5 | 2 | Colonic TET | 1 | Yes | No | / |
| 11 | F | 60 | 180 | E3 | 8 | 2 | Colonic TET | 1 | Yes | Yes | / |
| 12 | M | 25 | 30 | E3 | 9 | 3 | Colonic TET | 1 | Yes | No | / |
| 13 | M | 69 | 120 | E1 | 6 | 2 | Colonic TET | 2 | Yes | Yes | Yes |
| 14 | M | 82 | 120 | E2 | 7 | 2 | Colonic TET | 2 | No | No | Yes |
| 15 | M | 15 | 36 | E1 | 6 | 2 | Colonic TET | 2 | Yes | Yes | Yes |
| 16 | F | 27 | 24 | E2 | 7 | 3 | Colonic TET | 1 | No | No | / |
| 17 | M | 38 | 72 | E2 | 9 | 2 | Colonic TET | 2 | Yes | Yes | Yes |
| 18 | M | 42 | 96 | E2 | 8 | 2 | Colonic TET | 2 | Yes | Yes | / |
| 19 | F | 22 | 24 | E3 | 11 | 2 | Colonic TET | 2 | No | No | / |
| 20 | M | 51 | 24 | E2 | 7 | 3 | Colonic TET | 2 | No | No | / |
| 21 | M | 47 | 24 | E2 | 7 | 3 | Colonic TET | 1 | Yes | Yes | / |
| 22 | F | 49 | 60 | E2 | 7 | 2 | Colonic TET | 1 | Yes | Yes | Yes |
| 23 | M | 46 | 174 | E2 | 5 | 3 | Colonic TET | 2 | Yes | Yes | Yes |
| 24 | M | 34 | 8 | E1 | 5 | 2 | Colonic TET | 1 | No | No | / |
| 25 | M | 28 | 60 | E3 | 7 | 3 | Colonic TET | 2 | Yes | Yes | / |
| 26 | M | 57 | 60 | E1 | 5 | 3 | Colonic TET | 1 | Yes | Yes | Yes |
| 27 | M | 31 | 36 | E2 | 5 | 3 | Colonic TET | 2 | Yes | Yes | / |
| 28 | M | 53 | 60 | E2 | 6 | 3 | Colonic TET | 2 | No | No | / |
| 29 | F | 52 | 72 | E3 | 11 | 3 | Colonic TET | 3 | Yes | Yes | Yes |
| 30 | F | 53 | 36 | E2 | 7 | 3 | Colonic TET | 2 | Yes | Yes | / |
| 31 | F | 48 | 36 | E1 | 6 | 2 | Colonic TET | 3 | Yes | Yes | / |
| 32 | F | 40 | 48 | E2 | 8 | 3 | Colonic TET | 2 | Yes | Yes | Yes |
| 33 | M | 32 | 36 | E2 | 10 | 3 | Colonic TET | 1 | No | No | / |
| 34 | F | 58 | 132 | E2 | 8 | 2 | Colonic TET | 1 | Yes | Yes | / |
| 35 | F | 55 | 12 | E2 | 9 | 5 | Colonic TET | 4 | Yes | Yes | Yes |
| 36 | F | 57 | 24 | E2 | 8 | 2 | Colonic TET | 4 | Yes | No | Yes |
| 37 | F | 62 | 24 | E3 | 5 | 2 | Colonic TET | 4 | Yes | No | / |
| 38 | F | 49 | 360 | E2 | 7 | 3 | Colonic TET | 4 | Yes | Yes | / |
| 39 | M | 59 | 204 | E3 | 7 | 3 | Colonic TET | 4 | No | No | / |
| 40 | F | 45 | 60 | E1 | 6 | 3 | Colonic TET | 4 | Yes | Yes | / |
| 41 | M | 26 | 12 | E2 | 6 | 3 | Colonic TET | 4 | No | No | / |
| 42 | M | 36 | 12 | E3 | 9 | 3 | Colonic TET | 4 | No | No | / |
| 43 | F | 23 | 39 | E2 | 7 | 3 | Colonic TET | 2 | Yes | No | / |
| 44 | M | 50 | 6 | E2 | 10 | 3 | Colonic TET | 4 | Yes | No | / |
| 45 | M | 46 | 24 | E3 | 7 | 3 | Colonic TET | 4 | Yes | Yes | / |
| UC: ulcerative colitis; FMT: fecal microbiota transplantation; TET: transendoscopic enteral tubing. | | | | | | | | | | | |
