# Supplementary material for: Cross-Talk Between Butyric Acid and Gut Microbiota in Ulcerative Colitis Following Fecal Microbiota Transplantation
Source: Front Microbiol. 2021 Apr 12;12:658292. doi: 10.3389/fmicb.2021.658292 (PMC8071877; doi:10.3389/fmicb.2021.658292)
Supplement: Supplementary file 2 [file Data_Sheet_1.pdf]

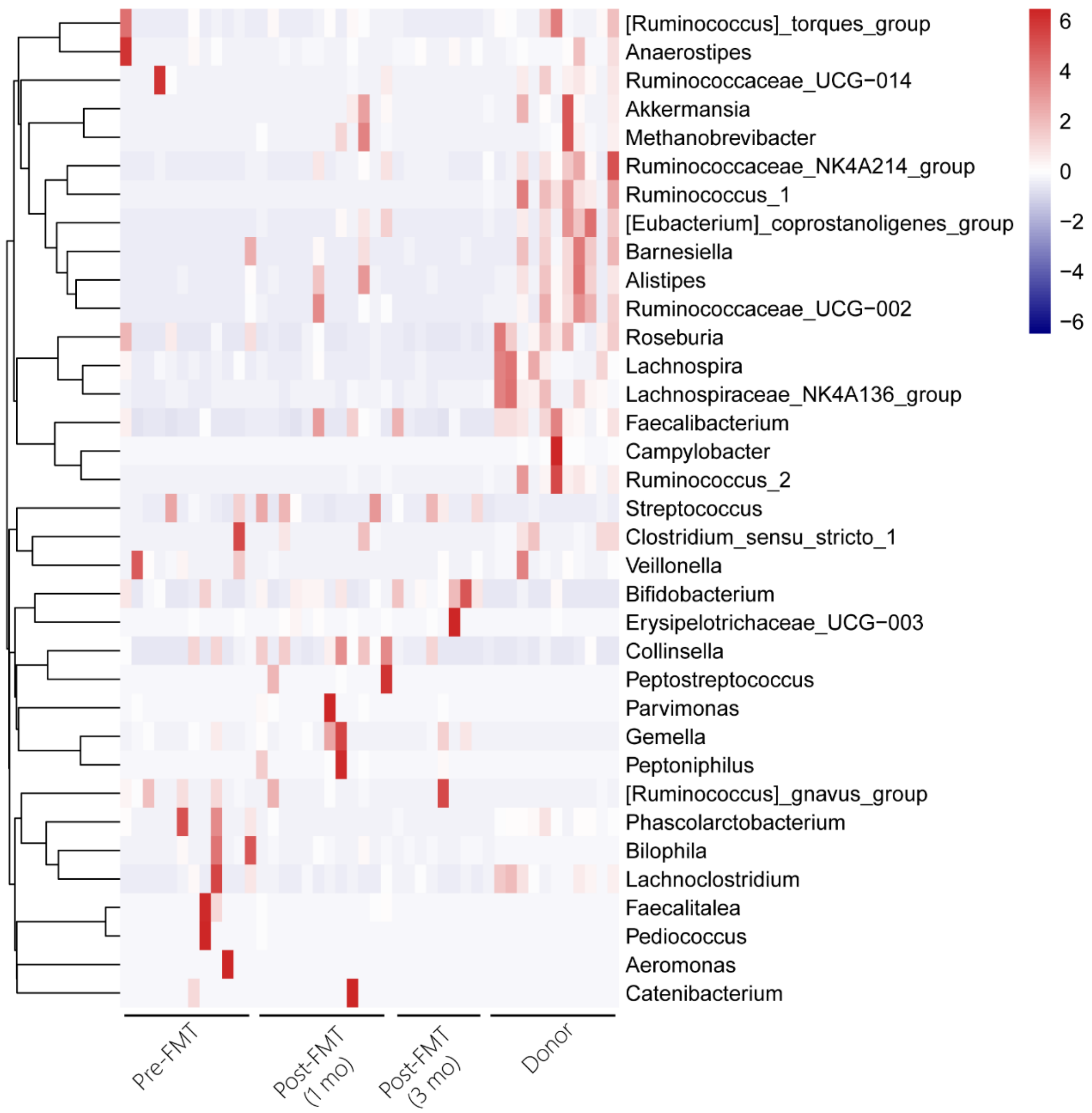

Supplementary Figure 1. FMT alters the abundance of fecal microbiota in UC patients and healthy donors at genus level. Fecal samples were collected before, one and three months post FMT and their microbiota, together with the donor samples were analyzed. Data are the heatmap analysis of the abundance of gut microbiota at genus level. Donor: Healthy donor.

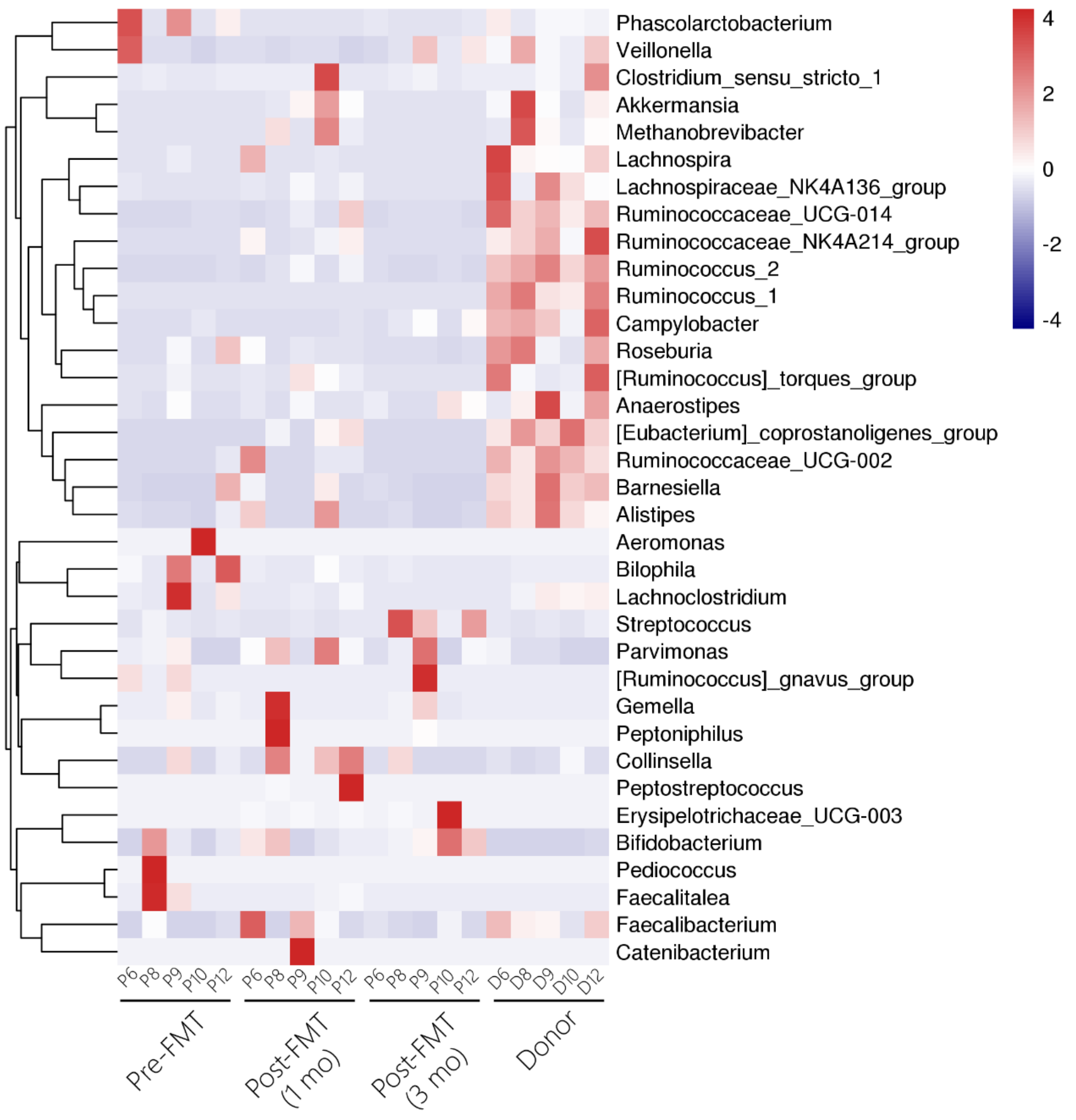

Supplementary Figure 2. FMT alters the abundance of fecal microbiota in UC patients and healthy donors at genus level. Fecal samples were collected before, one and three months post FMT and their microbiota, together with the donor samples were analyzed. Data are the heatmap analysis of the abundance of gut microbiota at genus level. Donor: Healthy donor. The specific patients were named as the numbers.

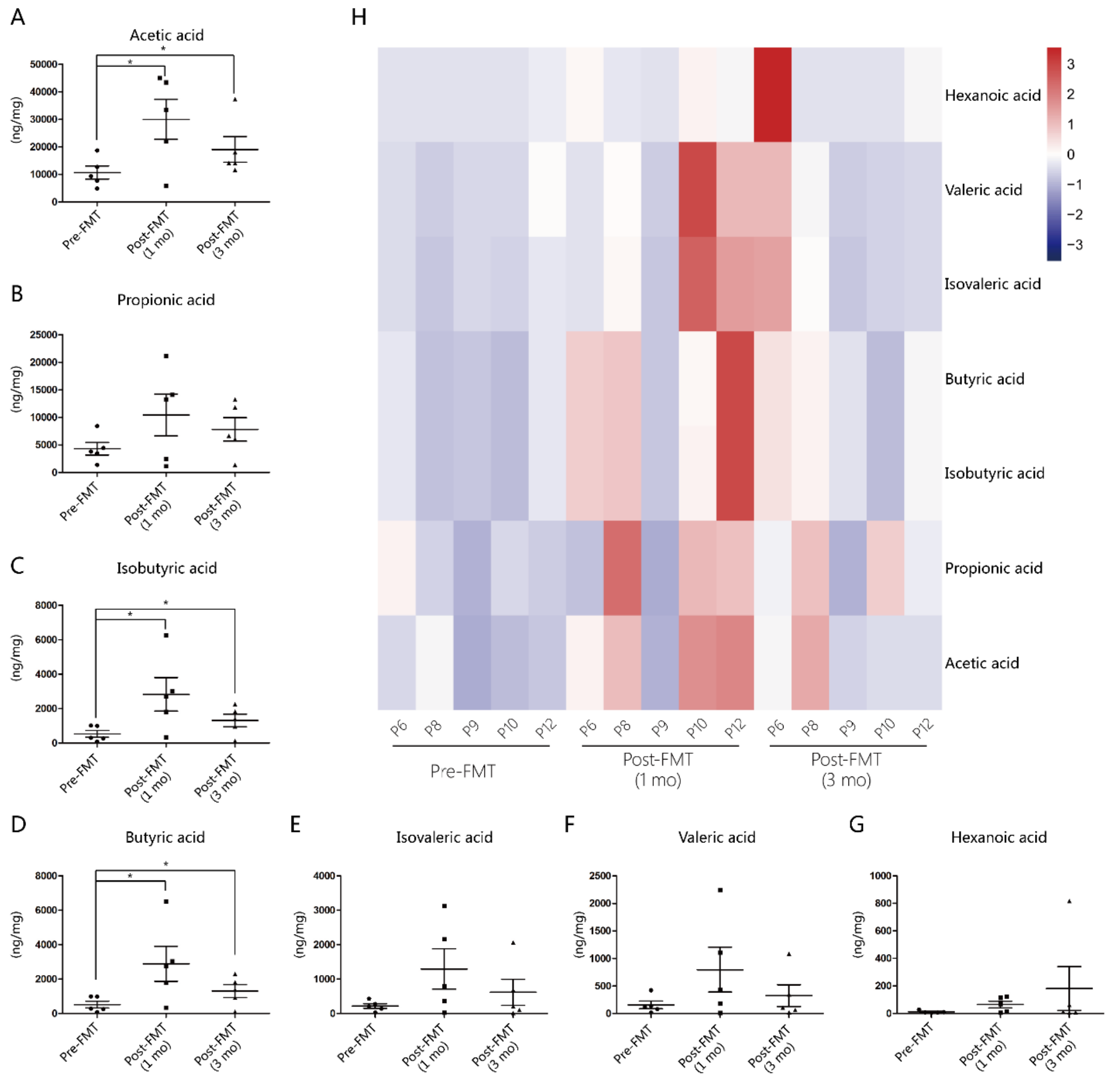

Supplementary Figure 3. FMT changes the contents of fecal SCFAs in UC patients.

(A-G) The levels of different types of SCFAs in individual patients. (H) Heatmap analysis of the dynamic changes in the levels of different SCFAs in individual patients. *$p<0.05$.
